# Integrating Citizen Scientist Data into the Surveillance System for Avian Influenza Virus, Taiwan

**DOI:** 10.3201/eid2901.220659

**Published:** 2023-01

**Authors:** Hong-Dar Isaac Wu, Ruey-Shing Lin, Wen-Han Hwang, Mei-Liang Huang, Bo-Jia Chen, Tseng-Chang Yen, Day-Yu Chao

**Affiliations:** National Chung Hsing University, Taichung, Taiwan (H.-D.I. Wu, W.-H. Hwang, M.-L. Huang, B.-J. Chen, T.-C. Yen, D.-Y. Chao);; Taiwan Endemic Species Research Institute, Jiji Town, Taiwan (R.S. Lin)

**Keywords:** avian influenza, citizen science, surveillance, risk map, spatial analysis, wild birds, viruses, Taiwan

## Abstract

The continuing circulation and reassortment with low-pathogenicity avian influenza Gs/Gd (goose/Guangdong/1996)-like avian influenza viruses (AIVs) has caused huge economic losses and raised public health concerns over the zoonotic potential. Virologic surveillance of wild birds has been suggested as part of a global AIV surveillance system. However, underreporting and biased selection of sampling sites has rendered gaining information about the transmission and evolution of highly pathogenic AIV problematic. We explored the use of the Citizen Scientist eBird database to elucidate the dynamic distribution of wild birds in Taiwan and their potential for AIV exchange with domestic poultry. Through the 2-stage analytical framework, we associated nonignorable risk with 10 species of wild birds with >100 significant positive results. We generated a risk map, which served as the guide for highly pathogenic AIV surveillance. Our methodologic blueprint has the potential to be incorporated into the global AIV surveillance system of wild birds.

Mapping the interface risk between wild birds and poultry requires information of wild bird distribution and migration patterns. Bird band recovery or global positioning system (GPS) tracking data are used for spatial risk mapping. Recently, citizen science data has become an increasingly valuable source for addressing a wide range of ecologic research questions. With this study, we provided the analytical framework of using eBird, a Citizen Scientist database (https://www.citizenscience.gov), to elucidate the dynamic distribution of wild birds and their potential for avian influenza virus (AIV) exchange with domestic poultry. We generated a risk map that can be integrated into the current AIV surveillance system, enabling strategic allocation of limited resources for spatially targeted virologic surveillance. The coding source, the open terrestrial environmental dataset, and eBird dataset are fully available at http://aiv.nchu.edu.tw.

AIV is an influenza A virus that belongs to the Orthomyxoviridae family. AIVs have been identified in a wide variety of species of wild and domestic birds, but their natural reservoir is wild waterbirds of the orders Anseriformes and Charadriiformes (e.g., ducks, geese, swans, and shorebirds). Wild waterbirds maintain a diverse group of low-pathogenicity avian influenza A viruses (LPAIVs), which cause limited illness in these host species (*1*). On the contrary, highly pathogenic influenza A viruses (HPAIVs), characterized by mortality of gallinaceous poultry, are limited to H5 or H7 subtypes and continue to cause illness and death in poultry worldwide (*2*,*3*). Periodically, human infections associated with HPAIV have been detected (*4*). In particular, the Eurasian (goose/Guangdong/1996 [Gs/Gd]) lineage has substantially affected global epizootic outbreaks of highly pathogenic avian influenza (HPAI), which have become enzootic in some areas and involve multiple waves of influenza with genetically distinct virus clades and subclades (*5*). Wild geese and ducks may form the bridge for AIV transmission between wild and domestic birds, which are kept alongside each other, creating the opportunity for genetic mixing of HPAIVs and LPAIVs when they infect the same bird concomitantly. Such genetic mixing promotes bidirectional virus exchange between wild and domestic birds for the continued adaptation of Gs/Gd HPAIVs in wild bird hosts and long-distance spread to new geographic regions along the flyway (*6*–*8*). Information about where wild and domestic birds can potentially interact on the landscape can help identify areas where disease transmission may be more likely to occur, useful for risk management and control measures. Such regions could become focal areas for surveillance and prevention (*9*).

The first step of mapping the interface risk requires information of wild bird distribution and migration patterns; however, obtaining such information is difficult. Without empirical data, previous studies implemented simulations or mathematical modeling for spatial risk mapping (*10*–*13*). Meanwhile, bird migration routes can be acquired from the bird band recovery (*14*) or GPS tracking data (*15*), but only a limited number of wild birds can be tracked and analyzed. Citizen science data are valuable for addressing a wide range of ecologic research questions, and the scope and volume of available data have rapidly increased globally (*16*). However, data from large-scale citizen science projects typically present a number of challenges that can inhibit robust ecologic inferences, including species bias, spatial bias, varied efforts, and varied observer skills (*17*–*19*). When using citizen science data, it is imperative to carefully consider the data processing and analytical procedures required to appropriately address the bias and variation.

Since 2015, Taiwan, which is on the East Asian Flyway of bird migration, has been affected by HPAI H5 virus clade 2.3.4.4, resulting in tremendous economic loss (*20*,*21*). In this study, we established an analytical framework (Figure 1) using citizen science data, eBird (*22*), to map the interface risk between wild birds and poultry flocks and to shed light on the underlying mechanism of AIV transmission in Taiwan. Our risk map presents a quantitative evaluation of the risk for AIV exchange at the interface between poultry flocks and wild birds, thereby enabling strategic allocation of limited resources for spatial targeting surveillance for AIV in wild birds and poultry.

**Figure 1:**
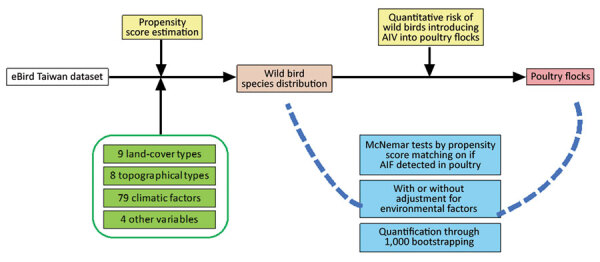
Framework of the analyses performed to map the risk of wild birds introducing avian influenza virus (AIV) into poultry farms for study of integrating citizen scientist data into the surveillance system for avian influenza virus, Taiwan.

## Materials and Methods

### Datasets and Software

We obtained bird-sighting records from the eBird Citizen Science database, the world’s largest citizen science program, providing fine-scale occurrence data of bird species (*23*). The reporting system is based on checklists (*22*), whereby the observer provides a list of birds detected, GPS location, sampling effort (whether all detected species are reported), sampling duration, sampling protocol (e.g., stationary point, travel, and banding and distance traveled in the case of traveling protocol), starting time of the sampling event, and number of observers. We used the eBird Taiwan dataset focusing on the records from January 2015 through June 2020. The Taiwan Endemic Species Research Institute, Council of Agriculture, Taiwan, established an open terrestrial environmental dataset with 1-km high resolution spanning 5-decade periods during 1970–2020 and used it to predict occupancy probability of the selected wild bird species (*24*). This dataset contains 100 variables, including 9 land-cover types (e.g., farmland, forest, or wetland), 8 topographies (e.g., latitude or slope), 79 climates (e.g., monthly average temperature or rainfall), and 4 other variables (e.g., traffic or length of roads). From the Council of Agriculture, Taiwan, we obtained the complete poultry farm census dataset, established in 2017 and based on an islandwide survey that used remote satellite imaging technology conducted by the Taiwan Agriculture Research Institute. The poultry farm outbreaks dataset was obtained from the surveillance system established by the Bureau of Animal and Plant Health Inspection and Quarantine, Taiwan, as described previously (*20*,*25*). During 2015–2017, a total of 1,223 poultry farm outbreaks were reported and laboratory confirmed in Taiwan (1,003 outbreak poultry farms in 2015, 38 in 2016, and 182 in 2017).

We partitioned Taiwan into 4,762 squares, each 3 × 3 km, consisting of 306 grids, covering the coastline for follow-up spatial modeling. We performed all graphs and statistics in R software (The R Foundation for Statistical Computing, https://www.R-project.org) and produced maps by using QGIS (http://www.qgis.org). The packages used in R can be found from coding sources provided at http://aiv.nchu.edu.tw/Open_data.html.

### Spatial Exploration

To explore the spatial relationship of land-cover types or wild bird distribution, we subjected the area of each land-cover type or propensity score from each grid estimated for individual wild bird species from the wild bird species distribution map to principal component analysis and t-distributed stochastic neighbor embedding (tSNE) analysis. The tSNE analysis is a modern dimension reduction method that uses an iterative algorithm to visualize the high-dimensional data in 2 dimensions while also revealing some global structures (i.e., clusters) (*26*).

### Wild Bird Species Distribution Map

To investigate the risk for AIV exchange at the interface between poultry flocks and wild birds, we first mapped the potential distribution of the wild bird species (Figure 1). All spatial models are based on partitions, which generated 4,762 grids, 3 × 3 km each. To eliminate spatial counting bias in eBird data, we applied a set of autoregressive logistic models to the eBird Taiwan dataset to estimate the occupancy probability of the distribution of each species of wild bird in each spatial grid (*27*) (Appendix 1). Because multicollinearity might be present, to improve the stability of regression estimation, we used the elastic net method for variable selection. If the zero-inflated Poisson model did not fit the data well, we used the zero-inflated negative binomial regression model instead (*28*,*29*). Last, we used the occupancy probability of each bird species for individual grids to generate the distribution map for individual bird species. The estimated probability of occupancy is the propensity score, which we used for the matched-pair design (*30*).

### Risk Mapping at the Interface of Wild Birds and Poultry

A fundamental problem with mapping the risk for AIV transmission at the interface between wild birds and poultry is the difficulty of quantifying the amount of contact between them. Hence, we measured relative spatial risk on a 3-km × 3-km grid by matching on the propensity score the occupancy probability (P_m_) of each bird species. The tolerance of matching criterion is P_m _× 10%, which means if the case grid has its estimated score , the matched control should have a score lying within the tolerance interval (P_m,l_,P_m,u_), in which P_m,l_ = 0.90 and P_m,u _= min(1.10, 1). We considered the approach of matched-pair design, in which the case grid contains >1 poultry farm outbreak and the control grid contains poultry farms with no outbreaks during 2015–2017. Because the species of wild bird is both itself a risk factor as well as a confounder, propensity scores for each species with respect to all other species are matched out. By this manner, we estimated the partial effect of that particular species, possibly with adjustment for environmental and terrestrial factors. The association was measured by the McNemar χ^2^ statistic on 1 degree of freedom. Because there could be many candidate controls for each case grid, we performed 1,000 bootstrapped resamplings to produce 1,000 -realizations under the null hypothesis that the specific species of bird has no association with the outbreaks. We report the bootstrap results using the notations N_pa _= number of positive associations in 1,000 replicates and N_sp _= number of significant positive associations in those N_pa_ experiments (Table). The proportion of N_sp_ can be interpreted as parallel to the concept of p value, if the complement of (1 – N_sp_/1,000) is taken. The proportion of N_sp_/1,000 reflects the strength against the null hypothesis; higher values imply stronger evidence. However, we did not adopt a strict criterion for statistical significance; that is, we did not require p to be <0.05 (Appendix 1). We used the proportion of N_sp_ as the probability of AIV being introduced by the wild birds into poultry farms or vice versa.

**Table:** Risk for avian influenza virus transmission from wild birds to poultry, with and without adjustments for environmental and terrestrial factors*

Wild bird species			Without adjustment			With adjustment	
Scientific name	Common name		N_pa_	N_sp_ (in N_pa_)		N_pa_	N_sp_
*Calidris subminuta*	Long-toed stint		998	544		1,000	784(A)
*Chroicocephalus ridibundus*	Black-headed gull		1,000	896		1,000	482
*Tachybaptus ruficollis*	Little grebe		976	116		998	402
*Gallinago gallinago*	Common snipe		944	87		991	191
*Anas acuta*	Pintail duck		916	18		980	174
*Pluvialis fulva*	Pacific golden plover		987	171		993	157
*Himantopus himantopus*	Black-winged stilt		968	138		816	27
*Sternula albifrons*	Little tern		999	416		964	9
*Hirundo rustica*	House swallow		956	119		X	X
*Bubulcus ibis*	Cattle egret		972	188		X	X

*N_pa_, no. positive associations in 1,000 replicates; N_sp_, no. significant positive associations in the N_pa_ experiments.

After matched-pair McNemar analysis, we used only the bird species with positive association to depict a risk map of AIV exchange at the interface between poultry flocks and wild birds. The risk, defined as an infection probability (R_j_) of grid j, can be estimated by an additive-multiplicative risk model (Appendix 1).

## Results

**Figure 2:**
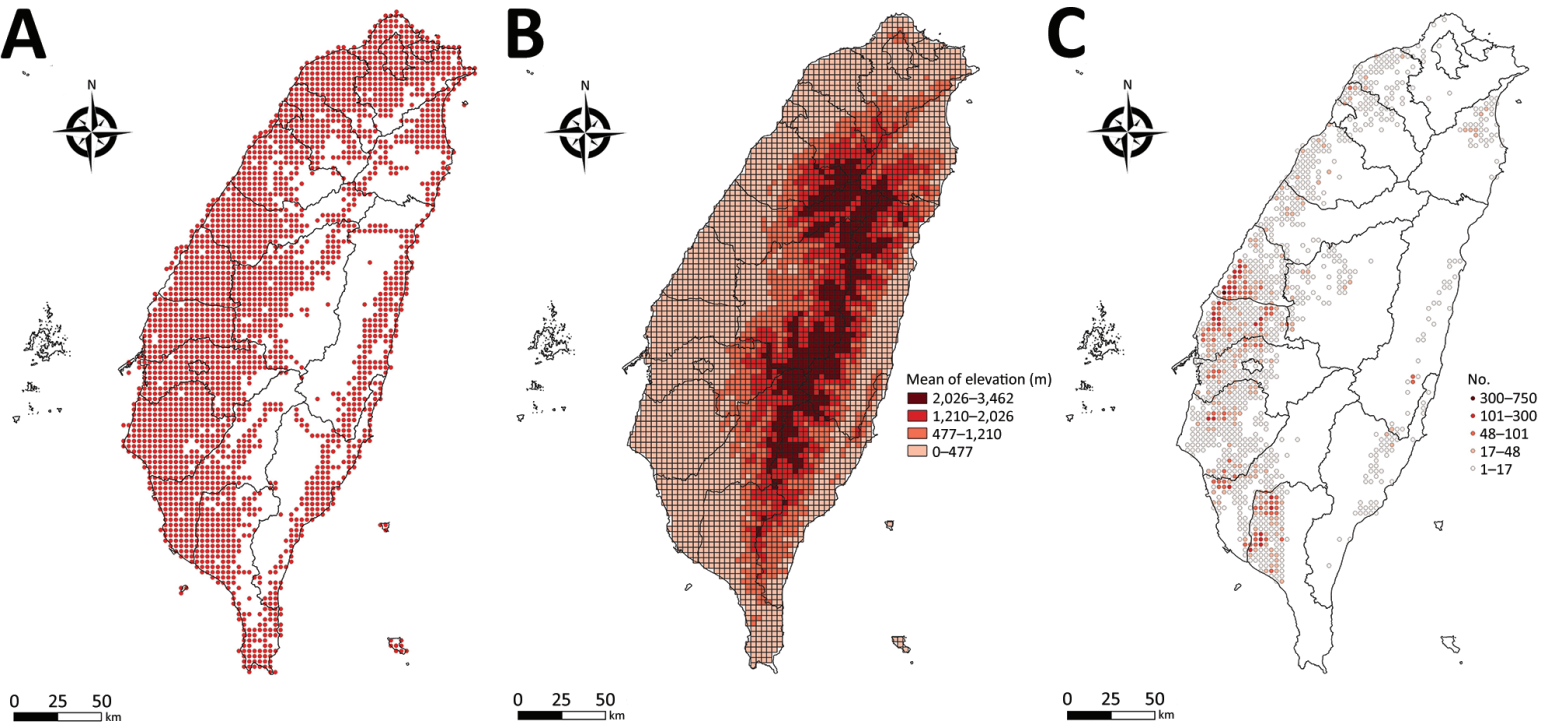
Distribution maps for study of integrating citizen scientist data into the surveillance system for avian influenza virus, Taiwan. A) The 3-km × 3-km grid with bird-sighting records based on Taiwan eBird dataset during 2015–2020; B) average altitude based on Taiwan open terrestrial environmental dataset; C) poultry farm census data for Taiwan.

We report all grids with bird-sighting records for 2015–2020 (Figure 2, panel A). There are no records for central grids of Taiwan because they are high-mountain areas and are not easily accessible by bird sighters. Because poultry farms are not distributed in the high-mountain areas (Figure 2, panels B, C), such sparse data did not affect our follow-up analysis.

**Figure 3:**
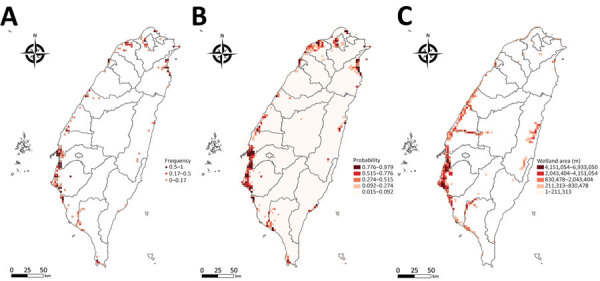
Distribution maps of pintail duck (*Anas acuta*) for study of integrating citizen scientist data into the surveillance system for avian influenza virus, Taiwan. A) True observation frequency from Taiwan eBird dataset; B) occupancy probability estimated by zero-inflated Poisson model; C) distribution map of wetland, based on the land-cover type from the Taiwan open terrestrial environmental dataset.

Occupancy probability was estimated by zero-inflated Poisson model (Figure 3, panel B). The distribution of the predicted occupancy probability is consistent with the bird-sighting distribution from the observer records (Figure 3, panel A) and highly overlaps with the wetland land-cover type (Figure 3, panel C). Distribution maps for all 68 species of wild bird are shown at http://aiv.nchu.edu.tw/migrating_species.html. Among the 68 species, 66 selected for this study can be well modeled for their occupancy probabilities by using a zero-inflated Poisson model.

**Figure 4:**
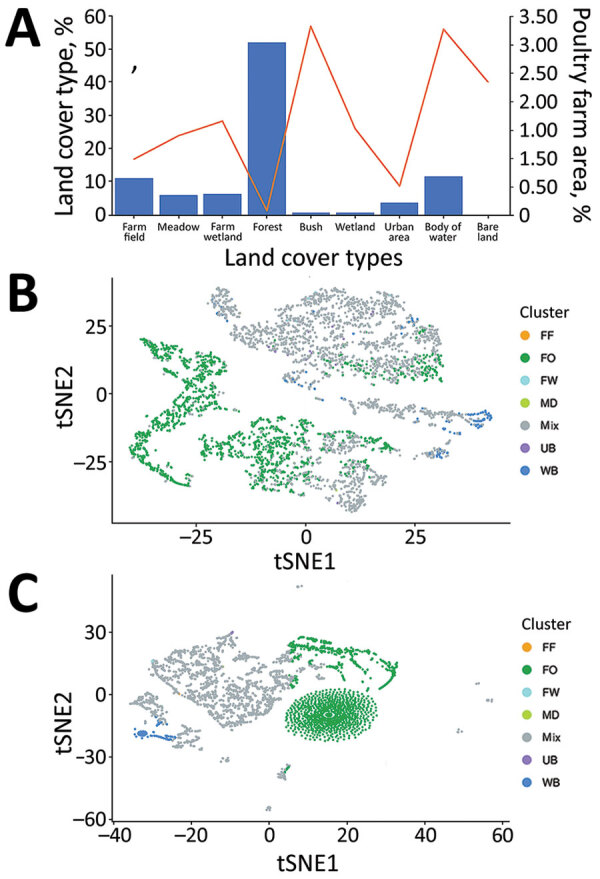
Land cover and bird distribution data for study of integrating citizen scientist data into the surveillance system for avian influenza virus, Taiwan. A) Percentages of 9 land-cover types in the total area of Taiwan main island (bars), area of poultry farms in the total area of indicated land-cover types (line). B, C) The clustering pattern of the area of each land-cover type (B) and the propensity score for each bird species from 3,764 grids partitioned by 3-km × 3-km squares of the main island of Taiwan (C), are based on principal component analysis and tSNE dimension reduction. Clusters are colored by the land-cover type as shown in panel A. For 4,762 grids, if 1 specific land-cover type is composed of >90% in the grid, such grid will be regarded as such specific land-cover type. Otherwise, it will be labeled as the mixed land-cover type. The labels of clusters in panels B and C are consistent with those in panel A. FF, farm field; FO, forest; FW, farm wetland; MD, meadow; mix, mixed land-cover types; tSNE, t-distributed stochastic neighbor embedding UB, urban; WB, water body.

The major land-cover type in Taiwan is forest, which comprises 55.8% of the total area of Taiwan’s main island, and <0.1% of the area is poultry farms (Figure 4, panel A). On the contrary, <2.5% of main island area is covered by bush, wetland, and bare land, which are the main land-cover types for poultry farming. Water bodies cover only 1.19% of the island but also contain 3.27% of the area for poultry farming, mainly Anseriformes, such as ducks and geese. Because the estimated occupancy probability of wild bird species is based on 4,762 grids, 3-km × 3-km, generated for the whole island, many grids are made of mixed land-cover types (Figure 4, panel B). To explore the relationship of wild bird distribution with land-cover type, the estimated occupancy probabilities, also referred to as propensity scores, of 68 different species of wild bird were also subjected to principal component analysis and tSNE. The results showed that various wild birds were distributed in different ecologic environments, including forest and bodies of water, for which probabilities for AIV exchange between poultry farms and wild birds might differ (Figure 4, panel C).

In the second stage of our analysis, we performed propensity score matching with bootstrapping to precisely map the probability of AIV exchange between poultry flocks and wild birds. By doing so, we treated environmental factors as confounders and included them for the purpose of multivariate adjustment. Through propensity score matching with the probability of wild bird appearance, the significance of poultry farm outbreaks caused by HPAIV could be examined by bootstrapped resampling scheme based on randomness in selecting case–control matched pairs. There were nonignorable species with *>*100 significant results among the 1,000 bootstrapped realizations of the McNemar statistic (Table 1). Four species of wild bird, including the long-toed stint, black-headed gull, little grebe, and pacific golden plover, were highly correlated with the HPAIV outbreaks on poultry farms, with or without adjustment. The wild bird species that can be viewed as being significant when environmental factors were considered, is the long-toed stint*,* with a p value of 0.216 (1 – 0.784) (Table 1). On the other hand, if environmental factors were not considered, the black-headed gull shows a highly significant association *(*p* = *1–0.896 = 0.104).

## Discussion

**Figure 5:**
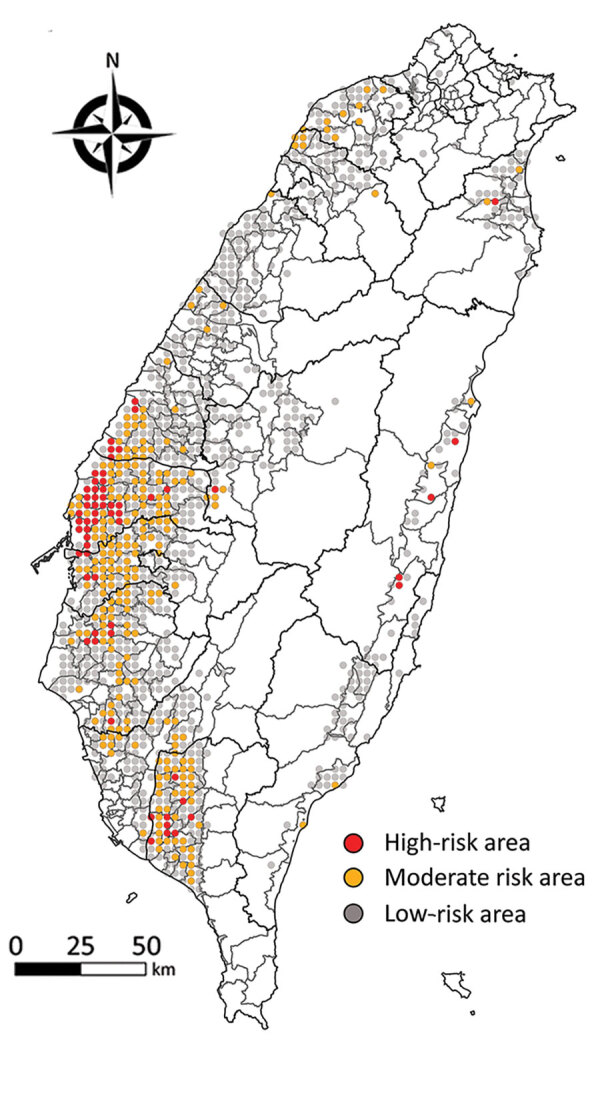
Risk maps showing risk of poultry farm acquiring avian influenza virus infection from migratory wild birds, from study of integrating citizen scientist data into the surveillance system for avian influenza virus, Taiwan. Each dot represents each 3-km × 3-km grid. Red dots represent the high-risk area with probability calculated based on 10 bird species with high risk of transmitting avian influenza virus into poultry farms (Table). Orange dots represent the middle-risk area, with bird species with >1 positive McNemar test result. Gray dots represent the low-risk area with bird species having no positive or negative McNemar test results.

The continuing circulation and reassortment of Gs/Gd-like HPAIV with LPAIV has caused huge economic losses and raised public health concerns because of its zoonotic potential (*31*). Virologic surveillance of wild birds has been suggested as part of a global AIV surveillance system (*32*,*33*) and could directly benefit human and animal health through knowledge of how avian influenza virus genes flow among different hosts and how factors that drive AIV prevalence in wild birds enable virus spillover, emergence, and maintenance. However, problems with understanding the transmission and evolution of HPAIV include underreporting, biased selection of sampling sites, and limiting AIV surveillance to wild bird carcasses (*34*). The risk map generated in this study (Figure 5) can be used for, but is not limited to, educational purposes of the government to communicate with stakeholders to increase their biosecurity of poultry farms; a sustained cost-effective AIV surveillance program that promotes sampling site selections, thereby enabling limited resources to be strategically allocated for early detection of changing AIV dynamics in reservoir populations to support public health and pandemic preparedness (*35*); and a quantitative assessment of the risk of introducing AIV from wild birds into poultry flocks as well as the possible transmission of AIVs between wild bird populations affected by bird behavior, age structures of populations, and detailed migration routes.

Pathogens that cross the interface between diverse populations, such as wildlife and livestock or animals and humans, pose particular challenges to developing effective and efficient surveillance and control measures. AIVs can spread globally among wild birds, poultry, and humans, with potentially devastating effects. The Citizen Science project eBird, which collects large volumes of data across broad spatial and temporal dimensions, provides a great opportunity for investigating how wild birds contribute to this spread. However, citizen science data often suffer from bias arising from bird sighters’ viewing preferences, convenience (for bird sighting and travel planning), incentives (if any), and others. It may even come from the process of data recording and reporting. Although different analytical approaches for minimizing the bias have been published (*36*–*38*), our study used a high-quality inventory filtering procedure by constraining aspects of the observation process (e.g., the duration of observation and records of bird species sighting by ignoring the counts of birds on the checklists to remove potential sources of variation and facilitate subsequent analysis). Furthermore, birds are observed mostly during the day, and wild birds may forage near waterfowl poultry farms during the night (*39*). Such foraging flight distance is relatively short (e.g., the median for pintail ducks marked with satellite transmitters is within 3 km) and is covered by the size of the grids here (C.-C. Chen, National Pingtung University of Science and Technology, pers. comm., 2022 Jul 1).

Another layer of bias in using the eBird dataset comes from the accessibility of bird sighting by the observers. Because the locations for bird sighting are highly influenced by the proximity to the road accessible by the observers, the distribution of bird-sighting records cannot fully reflect the ecologic distribution of the wild birds. However, the main difficulty with building a unified regression model to map the ecologic distribution of wild birds and using the eBird dataset is the number of variables from the open terrestrial environmental dataset. In our study, the numbers of bird species and environmental variables both exceed 100. Therefore, we focused on 1 species at a time but kept all other variables as confounders. The elastic net regularization method was first used as a unified machine learning algorithm to generate parsimonious models for estimating potential risk maps (*40*). The elastic net regularization method is a compromise between ridge regression and lasso regression. To avoid complexity, we modeled only the presence or absence of individual bird species in each grid by using a conditional autoregressive logistic model, taking spatial autocorrelations into account.

Wild waterfowl are known reservoirs for LPAIV and potentially HPAIV because of the global evolution and circulation of Gs/GD-derived clade 2.3.4.4 (*41*), which resulted in a new era of AIV surveillance requiring identification of critical interfaces between wild birds and poultry on the landscape for potential interspecies transmission and virus evolution. Although such estimates can be extrapolated from active poultry surveillance, as suggested by previous studies (*42*–*44*), accurately determining the likelihood (or potency) of the exchange of AIV at the interface between poultry flocks and wild birds is difficult because of incomplete active surveillance and a lack of biosecurity information for individual farms. In this study, we performed propensity score matching with bootstrapping by ensuring the randomness of case–control pair selections for estimating probability (*45*). By doing so, environmental factors were seen as confounders for which further adjustment can be made. We identified 10 nonignorable species of wild bird with >100 significant results among the 1,000 bootstrapped realizations of the McNemar statistic (Table 1). Among them, 4 wild bird species, including the long-toed stint, black-headed gull, little grebe, and pacific golden plover, were highly correlated with the introduction of HPAIV into poultry farms, with or without adjustment (Table 1). Those 4 species are mainly wintering birds; their preferred habitats are wetland or farmland. In particular, based on GISAID (https://www.gisaid.org), there are extensive records of LPAI in black-headed gull, little grebe, and pacific golden plover, which increases their chances of transmitting AIV into poultry farms as shown for the bootstrapping results (Table 1). Although the p values are not high, note that the term “p value” used here represents a concept of significance level based on bootstrapped samples, rather than the 0.05 level of significance criterion traditionally pursued in statistics.

The key limitation of our study is the lack of detailed information contributing to between-farm AIV transmission. Such information includes bridge bird species on or near poultry farms, transportation vehicles, or other farm animals (e.g., rats feeding on bird carcasses). It is also evident that different AIV subtypes and pathotypes can vary according to the epidemiology and prevalence of wild birds (*46*). For example, the following can interfere with significance results in McNemar tests: spatiotemporal variation in between-farm transmission by wild birds, species age structure, behaviors including roosting/breeding sites, AIV susceptibility, and AIV pathology. Although phylogenetic analysis of HPAIV from individual outbreak poultry farms could reveal between-farm transmission events, we, unfortunately, had no access to sequence data of outbreak viruses. We also selected 36 different nonmigratory wild birds and followed the same analytical frameworks as those for migratory birds. The results suggested that 4 nonmigratory wild bird species, including the black bulbul, black-headed munia, red collared dove, and common moorhen, could potentially serve as bridging species for introducing AIV into poultry farms (Appendix 2 Table 2), although other bridging species could also play major roles. Furthermore, increased occurrence of HPAI in wild birds resulted in disease and death of fairly large numbers of birds (>10,000 individuals) and affected diverse species (*47*). Mortality data for birds, especially nonmigratory species, could be indicators for HPAIV transmission and could be incorporated into spatiotemporal data analysis together with other genetic or bird behavior data in the future (*25*).

In summary, information about the spatial distribution of wild birds and how they exchange AIV with poultry, as well as the related risks, has the potential to benefit surveillance, pandemic preparedness, and prevention plans. However, poor availability of data presents challenges. The integration of citizen science data, such as eBird, into the surveillance system is underappreciated, and the workflow developed in our study can be applied in other countries for AIV surveillance in wild bird site selections to increase the breadth of virus strain coverage and knowledge of gene flow of AIV among wild birds.

Appendix 1 Supplementary methods for study of integration of citizen scientist data into the avian influenza virus surveillance system, Taiwan.

Appendix 2 Supplementary results for study of integration of citizen scientist data into the avian influenza virus surveillance system, Taiwan.
