# Supplementary material for: Integrating Citizen Scientist Data into the Surveillance System for Avian Influenza Virus, Taiwan
Source: Emerg Infect Dis. 2023 Jan;29(1):45–53. doi: 10.3201/eid2901.220659 (PMC9796195; doi:10.3201/eid2901.220659)
Supplement: Appendix 1 — Supplementary methods for study of integration of citizen scientist data into the avian influenza virus surveillance system, Taiwan. [file 22-0659-Techapp-s1.pdf]

Article DOI: https://doi.org/10.3201/eid2901.220659

Integration of Citizen Scientist Data into the Surveillance System for Avian Influenza Virus, Taiwan

Appendix

Part I: eBird dataset and wildbird species selection

Taiwan, on the East Asian route of bird migration, launched the eBird Taiwan program in 2015 and has since accumulated over 4,800 users by February 2022, who have contributed stable bird sighting checklists since 2015. After removing repeated checklists, a total of 336,154 checklists with 3,778,382 numbers of wild bird species recorded from each checklist were found in Taiwan the ebird dataset between January 2015 and June 2020, Multiple observations of the same birds can happen either because several observers travelled together or because they came independently to the same site on the same day, both situations creating pseudo-replication. Therefore, we only consider the presence or absence of wild bird species observed here.

To avoid reporting bias commonly found from citizen science dataset, we filtered the dataset with the three different criteria to obtain high-quality checklists comparable in amounts of efforts. The criteria include: (i) the traveling distance was less than 2 kilometers, otherwise they may not represent the local bird composition around the reported GPS location (*1*), (ii) the observation area was less than 100 hectares to ensure the identified bird species fell into 3km×3km grids, and (iii) the duration of continuous observation was limited to ≤240 minutes

since the duration exceed this criterion tends to correlate with particular bird sighting activity, such as Taiwan New Year bird count event (*2*). We didn't restrict the checklists based on the sampling protocol of the observers used since we are trying to capture all bird sighting activities regardless of whether the observers would record all species or target only specific bird species. After data filtering, we obtained the final dataset used for the analysis, which consisted of 2,366,327 records of total numbers of species, covering 735 species, from 3080 observers.

Wildbird species selection

Before constructing the wild bird distribution map, the initial step is the selection of wild bird species relevant for the introduction of either HPAI or LPAI into the poultry farm. The bird species which show passage or regularly occurring breeding and wintering with preference to areas in Taiwan, and passing once or twice a year, may potentially act as a reservoir for LPAI, and will thus be considered for selection. The final inclusion criteria of bird species was based on either the top 20% abundancy by ranking the counts from the checklists of the observers (*3*) or the influenza virus isolation records from 3 databases: Influenza Virus Database-NCBI (https://www.ncbi.nlm.nih.gov/genomes/FLU/Database/nph-select.cgi), EMPRES-I (https://empres-i.apps.fao.org/) and Influenza Research Database (IRD) (https://www.fludb.org/brc/home.spg?decorator=influenza) before the date of 12/03/2020. In total, 68 species of wild birds were included in this study, including 22 species selected which are ranked on the top 20% observations with a minimum of >100,000 being defined as "substantially" abundant. Appendix 2 Table 1 (https://wwwnc.cdc.gov/EID/article/29/1/22-0659-App2.pdf) summarizes the complete list of bird species with their scientific name and common names under international taxonomy based on the second edition of the Avifauna of Taiwan or Avibase (https://avibase.bsc-eoc.org/avibase.jsp).

Part II: Estimating the occupancy risk map

Variable selection

The main difficulty in building a universally valid regression is that the presence (or absence) of bird species involves many variables (including land-related factors and environmental variables). It is challenging to obtain a unified explanation about the model structure. To estimate a risk map (occupancy probability), a variable selection procedure, called the *elastic net* method, were used for screening significant variables, including bird species and environmental factors. The elastic net method is a compromise between ridge regression and Lasso (*4*,*5*).

Defining the resolution: 3km×3km grids

Let Taiwan be divided into a set of 3km×3 km grids, each with an area equal to 9 km^2, with four sides parallel to the Earth's longitudes and latitudes. This partition gives 4,762 grids covering the entire map of Taiwan including the coastline. Let the squares be denoted as $A_1^*, A_2^*, \ldots, A_{N^*}^*$ (N^*=4,762). Because not all $\{A_i^*\}_{i=1}^{N^*}$(denoted as $\mathcal{A}^*$) include both bird observations and poultry farms, let $\mathcal{A} = \{A_i\}_{i=1}^{N}$ denote a subset of $\{A_i^*\}_{i=1}^{N^*}$, where $\mathcal{A}$ includes only those with both farms and birds observation records (N=1,073). Hereafter we call $\mathcal{A}$ *the matrix of grids with bird observations*. Note that the grids in $\mathcal{A}^*\backslash\mathcal{A}$ that contain no poultry farms are the ones located at or near elevated mountain areas. Let ${y_{i,k}}^*$ be the number of birds of species k reported in the i-th grid; $y_{i,k} = \mathbf{1}\{{y_{i,k}}^* \geq 1\}$ is the indicator of whether there is any observation of k-species in that i-th grid; k=1,…,K with K being the total number of species. Further, let $t_{i,k,s}$be the value of the s-th variable for temporal, terrestrial, and environmental factors.

Modeling the occupancy

For species k, we first estimate the probability of its occurrence based on the presence or absence of other species as explanatory variables. This probability, denoted U and interpreted as a propensity score, is used as the "matching variable" in the following text. To model outbreaks in grids containing a certain number of poultry farms, the presence or absence of species k was used as the primary explanatory variable when the corresponding propensity scores U were matched. Therefore, it is still necessary to estimate the probability of occurrence of each species of bird in each grid based on a logistic autoregressive model to present an overall risk map.

Notations and model description

For grid "i" and for bird species "k", $Y_{i,k}$ is the indicator variable of existence of species "k", and $Y_{i,-k}$ is the indicator of all other species than species k. A natural conclusion is: the existence of species k depends on all the other species; and thus $Y_{i,-k}$ is a (K-1)-dimensional vector. Besides, $T_{i,k}$ is the vector-valued variable representing all other variables (including the environmental data) except for bird species. Explicit modeling of spatial correlation between Y and the other Ts is implemented through the variable $Y_{-i,k}$, which is also an indicator variable of observing species k in all adjacent grids using Queen's contiguity-based neighbors (*6*), that is $Y_{-i,k} = 1$ (*1*), where **A** is the event of $\sum_{\{-i\}} Y_{i,k} \geq 1$ when summed around grid "i", denoted by the set $\{-i\}$.

The ZIP model estimates the probability of bird occupancy in a grid that accommodates both structural zeros (species never appear in the grid) and random zeros:

$$\log(\lambda_{i,k}) = \beta_0 + Y_{i,-k}'\beta + T_{i,k}'\gamma + \varphi Y_{-i,k}, \text{ (A1)}$$

$$\log\left(\frac{\alpha_{i,k}}{1-\alpha_{i,k}}\right) = \theta_0 + Y_{i,-k}'\nu. \text{ (A2)}$$

Estimating occupancy probabilities using autoregressive logistic model

The principle of incorporating spatial autocorrelation is to consider the correlation with adjacent grids. Let $P(Y_{i,k} = 1|\mathrm{Y}_{\mathrm{i},-\mathrm{k}}, \mathrm{T}_{\mathrm{i,k}}, \mathrm{Y}_{-\mathrm{i,k}})$ be the occupancy probability given the "status" of the adjacent grids and the other land-cover and environmental variables. Explicit modeling of spatial correlation between Y and the other T is implemented through the variable $\mathrm{Y}_{\text{-i,k}}$ which is an indicator of observing species k in all adjacent grids (using Queen's contiguity-based neighbors).

$$\log\left(\frac{\mathrm{P}(\mathrm{Y}_{\mathrm{i,k}} = 1|\mathrm{Y}_{\mathrm{i},-\mathrm{k}}, \mathrm{T}_{\mathrm{i,k}}, \mathrm{Y}_{-\mathrm{i,k}})}{1-\mathrm{P}(\mathrm{Y}_{\mathrm{i,k}} = 1|\mathrm{Y}_{\mathrm{i},-\mathrm{k}}, \mathrm{T}_{\mathrm{i,k}}, \mathrm{Y}_{-\mathrm{i,k}})}\right) = \beta_0 + \mathrm{Y}_{\mathrm{i},-\mathrm{k}}{}'\beta + \mathrm{T}_{\mathrm{i,k}}{}'\gamma + \varphi\mathrm{Y}_{-\mathrm{i,k}} \quad \text{(A3)}$$

The occupancy probability is estimated through a ZIP model by summing the probabilities of nonzero terms:

$$\mathrm{P}(y_{i,k} = 0) = (1 - \alpha_{i,k}) + \alpha_{i,k}e^{-\lambda_{i,k}} \quad \text{(A4)}$$

In (A3), the advantage of using the indicator metric $\mathrm{Y}_{\mathrm{i},-\mathrm{k}}$ to model occupancy is that it avoids possible biases based on intrinsic properties of the eBird data, which can arise when reporting the number of species observed, but less often happens when only occupancy "status" is adopted. However, reducing bias inevitably leads to a loss of efficiency in statistical estimation. In the event the ZIP model is not suitable for model fitting, the zero-inflated negative binomial (ZINB) model can be used instead (*7*).

Propensity score and matched-pair design

The propensity score (U) corresponding to an indicator variable *Z*, which is random but dependent on a set of covariates, is the (estimated) probability of being equal to 1 for *Z*. To the purpose of adjustment for multiple explanatory variables (denoted by **X**) in this study, we consider **X** to include the indicators of observing species other than bird species k, as well as

many other variables. After the adjustment (for the propensity score), the risk factors are re-assessed for their association with the outcome variable (Y) by matching on the propensity score (*8*). The remaining question is: why use propensity score matching? First, the variable number of bird species can be large, making it impractical to report the risk of individual bird species one-by-one. Because of this concern, we consider the approach of matched-pair design so that the propensity scores of each species relative to all other species are matched. In addition, through this setting, the adjustment of environmental factors can also be achieved.

How to construct the case-control set

Among the N=1,073 grids where bird observations were reported, there are D=296 grids which contains at least 1 outbreak. We call the grid in D a "case", and for the other C=N-D=777 grids where no outbreak was reported, we call them "controls". In the sequel, we denote S_D as the set of "case" grids, and S_C as the set of "control" grids. Since every case can have multiple matched controls, it is possible to consider a resampling scheme from the matched control set and compute the McNemar statistic for each resampling.

McNemar's matched-pair association test and Bootstrapping

For a cell in S_D, and according to the *propensity* of each bird species estimated in the aforementioned grid, we use U_d to represent the potential of this grid according to a certain ordering method, d=1,..,,D. We look for matched control for each case grid in the following manner: Let U_c represent the propensity of the bird species calculated by the grid in the control set S_C, then $M_{c(d)}=(P_{m,l},P_{m,u})$, where $P_{m,l}$ and $P_{m,u}$ are stated in the "Materials and Methods" Section.

In the Appendix Table, let $\vartheta_d^{(k)} = 1$ if there is an observation record of the k-th bird species in at least one grid in S_D; otherwise $\vartheta_d^{(k)} = 0$. On the other hand, for the control grid

randomly selected out of the corresponding S_C subset $M_{c(d)}$, if this grid has an observation record of the k-th species, then $\vartheta_{c(d)}^{(k)} = 1$; otherwise $\vartheta_{c(d)}^{(k)} = 0$。

Appendix 1 Table. Forming McNemar chi-square tests from a matched-pair 2 by 2 table.

Condition (ii)	Condition (i) ➔ ⬇	A cell randomly selected from $M_{c(d)}$ has species k ? Yes	No
Cell d in S_D has species k	Yes	$\vartheta_d^{(k)} \times \vartheta_{c(d)}^{(k)}$	$\vartheta_d^{(k)} \times (1 - \vartheta_{c(d)}^{(k)})$
	No	$(1 - \vartheta_d^{(k)}) \times \vartheta_{c(d)}^{(k)}$	$(1 - \vartheta_d^{(k)}) \times (1 - \vartheta_{c(d)}^{(k)})$

In these four yes-no cells, only one of them equals to 1, the other three equal to 0. Here a "one" represents "one matched-pair". Taking the summation over d=1,…,D, we obtain the total number of discordant pairs. Further, let $\alpha^{(k)}$ be the number of pairs that the case grid has species-k but the control-grid does not;

$$\alpha^{(k)} = \sum_{d=1}^{D} \vartheta_d^{(k)} \times (1 - \vartheta_{c(d)}^{(k)}),\ \beta^{(k)} = \sum_{d=1}^{D} (1 - \vartheta_d^{(k)}) \times \vartheta_{c(d)}^{(k)}. \text{ (A5)}$$

Conversely, $\beta^{(k)}$ is the number of pairs that the case-grid has no species-k but the control-grid does have. Because the random sampling is implemented on $M_{c(d)}$, a subset of S_C, the bootstrapping suggests that this random sampling can be repeated B times for a large "B" (*9*). If, temporarily, the number of poultry farm in the cells are not taken into account (but actually the number itself is a risk factor of the outbreak indicator of that cell), denote $\alpha_b^{(k)}$ and $\beta_b^{(k)}$ to be the numbers of discordant pairs with conditions (i) and (ii) stated above for species k, and at the b-th resampling. Let $\chi_b^{(k)}$ be the realization of McNemar statistic calculated at the b-th resampling:

$$\chi_b^{(k)} = \frac{(|\alpha_b^{(k)} - \beta_b^{(k)}| - 1)^2}{\alpha_b^{(k)} + \beta_b^{(k)}},\ b=1,2,\ldots,B \text{ (A6)}$$

The McNemar statistic in (A6) only provides a measure of significance, so we need to further consider the issue of positive or negative association.

Let $\text{sign}(\alpha_b^{(k)} - \beta_b^{(k)})$ denote the indicator of positive or negative association between the k-th species and the outbreak event. Using $1_{\{\alpha_b^{(k)} > \beta_b^{(k)}\}}$ and $1_{\{\alpha_b^{(k)} \leq \beta_b^{(k)}\}}$ to represent the indicator of positive or negative association, respectively, in the b-th replication of the bootstrapping procedure, we have (for b=1 to B):

$$p^{(k)} = \frac{1}{B}\sum_{b=1}^{B} 1_{\{\alpha_b^{(k)} > \beta_b^{(k)}\}},\ q^{(k)} = \frac{1}{B}\sum_{b=1}^{B} 1_{\{\alpha_b^{(k)} \leq \beta_b^{(k)}\}} = 1 - p^{(k)}. \text{ (A7)}$$

The quantities $p^{(k)}$ and $q^{(k)}$ measure the tendency of positive and negative associations, respectively, using the resampling procedure.

Risk map of AIV introduced into poultry farm by wild birds

After matched-pair McNemar analysis, only the bird species with positive association were used to depict a risk map of AIV introduced into poultry farms by wild birds. The risk, defined as an infection probability (R_j), of grid j can be estimated by an *additive-multiplicative* (AM) *risk model* through the decomposition:

$\widehat{R}_j$=Pr(appearance of birds species)*

Pr(introduction of AIV to poultry in grid j|appearance of bird species)*

Pr{proportion of poultry farms in area in grid j}*

Pr{a poultry farm infected by HPAIV} (*1*) The first two terms are estimated, joined by $\frac{\sum_k S_k I_{jk}}{K \times B}$, where the quantities $\{S_k\}$ are the numbers of positively significant association (for species k) in the "B" bootstrapped re-samplings; obviously, $\frac{S_k}{B}$ offers a bootstrap estimate for the "gravity level" of significance, and $\{I_{jk}\}$ are the propensity scores estimated for species k. Let A_j be the number of outbreak poultry farms, D_j be the total number of poultry farms and F_j denotes the total area (in km^2) of poultry farms potentially to be infected in grid j. Therefore, the

probability of wild birds introducing AIV into poultry farms in grid j is estimated through the additive model as:

$$\widehat{R}_j = \frac{\sum_k S_k I_{jk}}{K \times B} \times \frac{A_j}{D_j} \times \frac{F_j}{9\ km^2}$$

This $(A_j / D_j) \times (F_j / 9\ km^2)$ can be treated as the proportion (probability) that a randomly selected poultry farm is an infected one in that grid.

References

1. Taiwan Endemic Species Research Institute (TESRI). Taiwan eBird waterfowls hotspots [cited 2020 Aug 5]. https://wwwtesrigovtw/A6_3/content/32539

2. Lin D, Lin Y, Chao J, Chang A, Pursner S, Lyu A, et al. Taiwan New Year Bird Count 2020 Annual Report. Taiwan Wild Bird Federation, Taiwan Endemic Species Research Institute, Taiwan. 2020.

3. Sullivana BL, Aycrigg JL, Barry JH, Bonney RE, Bruns N, Cooper CB, et al. The eBird enterprise: An integrated approach to development and application of citizen science. Biol Conserv. 2014;169:31–40. https://doi.org/10.1016/j.biocon.2013.11.003

4. Zou H, Hastie T. Regularization and variable selection via the elastic net. J R Stat Soc B. 2005;67:301–20. https://doi.org/10.1111/j.1467-9868.2005.00503.x

5. Tibshirani R. Regression shrinkage and selection via the lasso. J R Stat Soc B. 1996;58:267–88. https://doi.org/10.1111/j.2517-6161.1996.tb02080.x

6. Anselin L. Spatial econometrics: methods and models. Dordrecht: Kluwer Academic Publishers; 1988.

7. Greene W. Accounting for excess zeros and sample selection in Poisson and negative binomial regression models [cited 2022 Nov 22]. NYU Working Paper No EC-94-10. 1994. https://papers.ssrn.com/sol3/papers.cfm?abstract_id=1293115

8. Rosenbaum PR, Rubin DB. The central role of the propensity score in observational studies for causal effects. Biometrika. 1983;70:41–55. https://doi.org/10.1093/biomet/70.1.41

9. Efron B. Bootstrap Methods: Another Look at the Jackknife. Ann Stat. 1979;7:1–26. https://doi.org/10.1214/aos/1176344552
